# The Study of Cognitive Characteristics in Asperger’s Disorder by Using a Modified Prisoner’s Dilemma Game with a Variable Payoff Matrix

**DOI:** 10.1371/journal.pone.0048794

**Published:** 2012-11-07

**Authors:** Masaya Tayama, Masaru Tateno, Tae Woo Park, Wataru Ukai, Eri Hashimoto, Toshikazu Saito

**Affiliations:** 1 Department of Neuropsychiatry, Sapporo Medical University School of Medicine, Sapporo, Japan; 2 Department of Psychiatry, Boston University School of Medicine and Veterans Affairs Boston Healthcare System, Boston, Massachusetts, United States of America; Hungarian Academy of Sciences, Hungary

## Abstract

Individuals with Asperger’s Disorder (ASP) have difficulties in social reciprocity and in providing appropriate cooperative behavior. The Prisoner’s Dilemma (PD) is a well-known model in game theory that illustrates the paradoxical disposition of interaction between two individuals with opposing interests, and may be a useful tool in the diagnosis of ASP in early childhood. In this study, we investigated the cognitive characteristics of ASP by using a modified PD game. The subjects were 29 individuals with ASP and 28 age- and IQ-matched controls. In the PD game, each of two players has two cards: card 1 represents cooperation and card 2 betrayal. The score each player obtains is decided according to a 2 x 2 payoff matrix and depends on the combination of their selections. The P-score (“P” for punishment) is defined as the score that is given when they both select betrayal. Comparing the two groups, the mean P-score at the end of the game and the mean total score were significantly higher in the ASP group, while the rate of selection of cooperative choice in both groups did not differ significantly. The classification of the shape of the graph according to fluctuation of the P-score revealed that in the ASP group only 2 cases (6.9%) showed continuous decrease of P-score compared to 8 control cases (28.6%) demonstrating similar results. However, the reasons were thought to be different: ASP subjects presumably selected card 2 because of a preference for the number itself, whereas control subjects preferentially chose this card to enhance their chance of winning the competition. It is often difficult to diagnose ASP in the young especially when they lack the distinctive clinical features of ASD in early childhood. Given the limited number of objective tools to evaluate the cognitive characteristics of ASP subjects, the PD game might be a useful diagnostic support tool for ASP.

## Introduction

Asperger’s Disorder (ASP) is one of the five subgroups of Pervasive Developmental Disorders (PDD) along with autistic disorder [1]. The essential features of ASP are severe impairment in reciprocal social interaction and the development of restricted, repetitive patterns of behavior, interests, and activities. To distinguish ASP from autistic disorder, no clinically significant general delay in language in early childhood must be confirmed in ASP. Recent epidemiological surveys revealed that the prevalence of PDD is almost 1% [2]–[5]. In Japan, epidemiological studies conducted in the late 1980s reported a higher prevalence rate of infantile autism compared to other countries [6]–[8]. Furthermore, a recent survey based on an integrated screening demonstrated that the incidence of PDD was 1.81% in Toyota, Japan [9]. The increased prevalence of PDD is a notable phenomenon worldwide [10].

Individuals with ASP have difficulties in social or emotional reciprocity, in providing appropriate sympathy and cooperative behaviors and in the use of multiple nonverbal behaviors such as eye-to eye contact, facial expression, and gestures to regulate social interaction. Because of these difficulties, patients with ASP can misinterpret feelings and intentions of others.

The concept of “theory of mind” was first proposed in 1978 by two scientists, Premack and Woodruff [11]. Theory of mind is the innate capacity which enables us to understand mental states behind other peoples’ outward behavior, such as beliefs intentions, feelings, hope and desires. It has been reported that persons with PDD are delayed in obtaining this ability compared to children without PDD [12], [13].

The Prisoner’s Dilemma (PD) is a well-known model in game theory [14]. PD illustrates the paradoxical disposition of interaction between two individuals with opposing interests. PD has applications in economics and business [15], [16]. It is frequently cited to describe the situations in which two persons choose different actions in an attempt to maximize their returns and as a result, often cause irrational results.

Many studies using the PD paradigm have been conducted [17], [18]. However, these previous studies have been concerned with various effects in the aspects of mixed-motive behavior, such as communication conditions [19], the payoff parameters of the game [20], and personality traits of the players [21]. In general, these previous studies omitted a dynamic payoff structure, an important dimension of conflict situations. Major attention has focused on the question of how an invariable payoff matrix affects the decision-making process of the players [22].

Recent neurocognitive studies have employed a game theory approach to explore the underlying mechanism of social dysfunction [23], [24]. In this respect, individuals with PDD could be optimal subjects for these studies because PDD is characterized by a significant impairment in social interaction. However, thus far, few studies with game theory-based methods have been conducted in PDD [25]–[28].

In this study, we developed a modified PD game with a variable payoff matrix and investigated the cognitive characteristics of ASP under the condition in which the payoffs are determined not only by their own immediate behaviors but also by the past history of their interaction in the game environment.

## Materials and Methods

### Subjects

The details of the subjects are summarized in Table 1. The subjects of this study were 29 adolescent individuals (male/female = 17/12) who visited the child and adolescent psychiatry-developmental clinic (age at first visit less than 18 years old) or neuropsychiatry clinic (one of the four staff psychiatrists is a child and adolescent psychiatrist) at Sapporo Medical University Hospital. To be included, subjects had to meet the following inclusion criteria: 1) age of 15 and over (old enough to enter a high school), 2) complete the Japanese version of Wechsler Adult Intelligence Scale (WAIS)-III, 3) be diagnosed with ASP based on DSM-IV-TR criteria, and 4) full-scale IQ (FSIQ) on WAIS-III of 85 or higher. Exclusion criteria included comorbid psychiatric disorders that could affect the results of the PD game (e.g. major depression, eating disorder, or obsessive-compulsive disorder) and the axis II diagnosis of mental retardation. Most subjects were referred to the clinics with the probable diagnosis of PDD by school counselors, school teachers, pediatricians, primary care physicians or general psychiatrists.

**Table 1:** Subject Characteristics.

	Asperger’s disorder	Normal control	*p*
n	29	28	
(Male/Female)	(17/12)	(18/10)	
Age	17.4±2.5	17.8±2.8	0.600
FSIQ	103.2±14.6	105.5±13.5	0.547
VIQ	106.1±15.8	106.2±14.5	0.971
PIQ	98.0±16.7	103.3±11.7	0.171

IQ: Intelligence Quotient, FSIQ: full-scale IQ on Wechsler Adult Intelligence Scale (WAIS)-III, VIQ: Verbal IQ on WAIS-III, PIQ: Performance IQ on WAIS-III.

The p values were obtained by Student’s t-test. No significant differences were found between the 2 groups. The age of each group was indicated as the mean ± S.D.

The normal control group consisted of 28 age- and IQ-matched subjects (male/female = 18/10). All control subjects underwent the Structured Clinical Interview for the DSM-IV Axis-I Diagnoses (SCID-I) and were interviewed by a certified child and adolescent psychiatrist [29] to exclude psychiatric or developmental disorders. Their intelligence levels were assessed by WAIS-III.

Regarding the required intelligence level for the present game, the results of our preliminary study revealed that FSIQ of 70 or higher would be necessary to understand the rules of this modified PD game completely. Since the subjects of this study were ASP and normal control, none of the subjects with FSIQ under 85 were included.

### The Modified Prisoner’s Dilemma (PD) Game

In the PD game, two players receive two cards: Card 1 = cooperation and Card 2 = betrayal. The score each player obtains is decided according to the payoff matrix as shown in Table 2. The 2×2 matrix makes four different score patterns. In the matrix, R stand for Reward for mutual cooperation, P for Punishment for mutual defection, T for Temptation to defect and S for Sucker’s payoff. In these conditions, when the matrix fulfills the formula 2R > S+T and T > R >P> S, the game can be defined as PD [30], [31].

**Table 2:** Canonical 2×2 PD Payoff Matrix.

	*Cooperate*	*Defect*
Cooperate	R, *R*	S, *T*
Defect	T, *S*	P, *P*

The 2×2 matrix based on the combination of both players’ selection makes four different patterns of scores. In this matrix, R stands for Reward for mutual cooperation, P for Punishment for mutual defection, T for Temptation to defect and S for Sucker’s payoff. In these conditions, when the matrix fulfills the formula 2R > S+T and T > R >P> S, the game can be defined as Prisoner’s Dilemma.

The payoff matrix used in this study is shown in Table 3. The structure of this matrix indicates that, from the viewpoint of player A, if player A selects Card 2, A’s points will always be higher or equal to that of player B. For example, if player A picks Card 2 and B selects Card 1, A gains 5 points and B loses 5, whereas if both A and B display Card 2, the score is equal at −4. Thus, to reach higher points together, both players need to cooperate for this purpose.

**Table 3:** The Payoff Matrix of This Study.

	B 1		B 2	
*A 1*	*4 (R)*	**4 (R)**	−*5 (S)*	**5 (T)**
*A 2*	*5 (T)*	−**5 (S)**	−*4 (P)*	−**4 (P)**

This 2×2 matrix indicates the score given to each player depending on the combination of their choices. The P-score, which goes to both players when they cooperate, at the beginning of the game was set as −4. The P-score changed according to the results of the previous trial. Scores in *italics* are given to player A and scores in **bold** are given to player B.

For the present study, we developed the PD game with a variable payoff matrix by following a procedure similar to the game proposed by Nakahara [32]. In the modified PD game for this study, the score for Punishment (P-score) becomes variable. By making the P-score changeable, we can add a variable factor to the conventional 2 x 2 payoff matrix for the ordinary PD game. As a result, if both players continue to display uncooperative behaviors, the situation will exacerbate, whereas as long as players A and B cooperate, both of them will be rewarded.

The P-score in this study was determined according to the following conditions:

1. The P-score at the beginning of the game was set as −4;
2. If both players indicate Card 1 at trial N, the P-score for trial N+1 increases by 1;
3. If both players indicate Card 2 at trial N, the P-score for trial N+1 decreases by 1.

In this context, the P-score can be regarded as the punishment for their uncooperative behavior, and the reduction of P-score could facilitate their cooperative behavior [33].

### The Procedure of the PD Game

Two players sat at the corner of a desk at a right angle holding two cards each, Card 1 (cooperation) and Card 2 (betrayal). A laptop was placed on the desk and the screen was visible to both players. The payoff matrix with the latest P-score was displayed on the laptop screen along with the total number of trials and the total score of each player. The game program for the computation of the P-score and the total score was made by the first author using Microsoft Visual Basic for Applications of Microsoft Excel 2007 (Microsoft Corporation, Washington, USA). After a prompt from the investigator, each player showed the selected card simultaneously. Then, the subject was requested to input the result, i.e. the combination of the selected number (11, 12, 21 or 22), to the game program using the ten-keypad. The correct input was double checked by the investigator to proceed to the next trial. The players are prohibited from communicating with each other during the game although they were sitting in close proximity to each other. In this study, the investigator served as player A and the subject was player B.

The game ended when one of the following three conditions was met:

1. The total number of trial reached 100;
2. The P-score reached +25;
3. The P-score reached −25.

Subjects were not notified of these conditions due to the chance that knowledge of these conditions could affect their decision-making, i.e. the subject would be prone to select Card 2 at the last trial [34], [35]. To avoid arousing a spirit of competition in the subject, the explanation prior to the game was simplified as much as possible. The game instructions included only the phrase ‘please make your card selection in order to increase your score’. To confirm understanding of the rules by the subject, several test trials were performed before data acquisition. The whole process of the PD game took approximately 30–40 minutes. Shortly after the game, the subject was interviewed by the investigator to understand his decision-making.

To avoid the effect of variable play strategy, only one investigator served as player A for all subjects. The investigator selected his card following a tit for tat (TFT) strategy which has been reported to be highly effective for the iterated PD. To make use of this strategy, the player begins the game by selecting cooperative choice, and then keeps picking the choice selected by the other player in the previous trial. For example, if the player B previously chose Card 1, then player A chose Card 1 for the next trial. Axelrod et al. [36] demonstrated that the TFT strategy was the most effective tactic in game theory for the iterated PD.

However, to help achieve the study aim of investigating the cooperative behaviors by the subjects with ASP, when the subject (player B) selected Card 2 eight consecutive times, the investigator (player A) intentionally increased presenting Card 1 to exaggerate his attitude of cooperation. If the subject still did not change his/her behavior responding to the cooperative choice, the investigator resumed the TFT strategy indefinitely.

### Statistical Analysis

Statistical analysis was performed using SPSS 16.0J for Windows (SPSS Japan Inc., Tokyo, Japan). Study results were expressed as mean ± SD. Two-group comparison was performed by Student’s t-test. The statistical significance was set at a p-value of less than 0.05.

### Ethical Matters

The study protocol was approved by the ethics committee of Sapporo Medical University. Informed written consent was obtained from all subjects prior to enrollment in the study. For subjects under 18 years of age, informed consent was obtained from their guardian as well as from the subjects themselves. All subjects participated in this study without any incentive. Similarly, all authors and subjects involved in this study declared themselves free of any conflict of interest relating to the study.

## Results

### 1. The P-score at the End of the Game, the Total Score, the Rate of Respective Selection Behaviors

The results are summarized in Table 4.

**Table 4:** The Results of the PD Game.

	Asperger’s Disorder	Normal Control	*p*
P-score at the end of the game^**^	10.97±14.0	−1.11±16.3	0.002
Total score^**^	204.76±168.5	36.21±218.3	0.001
Selection of Card 1 (%)	56.6±18.0	48.8±14.9	0.078
Rate of TFT (%)	57.8±18.3	53.2±11.9	0.274
Rate of concession^*^ (%)	16.0±8.2	20.9±8.3	0.028
Rate of defect (%)	26.0±12.4	25.8±8.6	0.920
		^*^ *p*<0.05 ^**^ *p*<0.01	

TFT: Tit for tat. The results are shown as mean ± S.D. The p values were obtained by Student’s t-test and considered significant when they were <0.05.

The selection behaviors of the subjects are categorized into 3 groups according to the following definitions:

Defect: player B chose Card 2 (betrayal) at the N trial, although the other player (player A) showed Card 1 at the N−1 trial.
Concession: player B chose Card 1 (cooperation) at the N trial, although the other player (player A) showed Card 2 at the N−1 trial.
TFT: At the N trial, player B chose the same card that the other player selected the previous (N−1) trial.

Both the P-score at the end of the game and the total score were significantly higher in the ASP group compared to the control. The rate of cooperative selection (the choice of Card 1) revealed no statistically significant difference between the 2 groups.

Regarding the pattern of card selection behavior, the rate of concession was significantly higher in the control group suggesting an attitude of cooperation with the other player.

### 2. Fluctuation of the P-score

**Figure 1:**
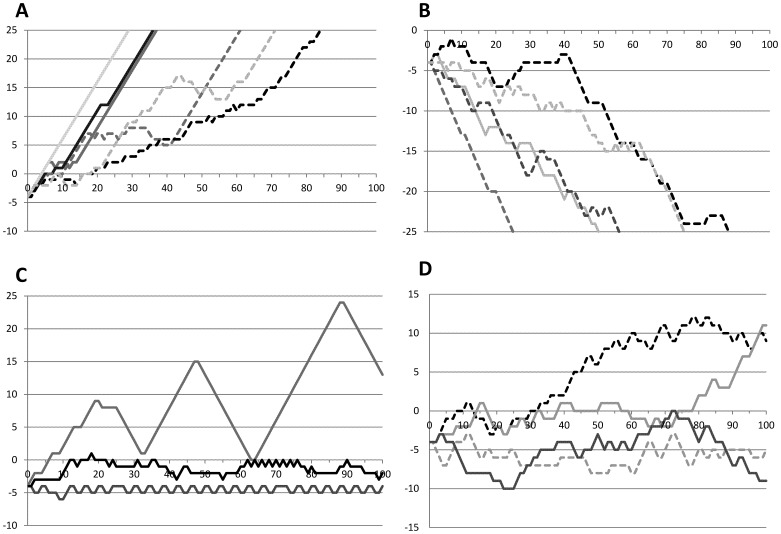
Graphs of the Fluctuation of the P-score Classified into 4 Groups. A: Upward type, B: Downward type, C: Repetition type, D: Unspecified type The solid lines represent the results of the ASP group and the broken lines that of the controls. The X axis represents the number of trial and the Y axis represents the P-score. Representative results of each group are shown in this figure. The difference in the depth of the color was given to make it easier for readers to distinguish each line.

The graphs showing the fluctuation in P-score are classified into four groups based on the shape of the graph and are described below. Typical results are shown in Figure 1.

Upward type: Games were classified as this type if the P-score kept increasing and the game ended when the P-score reached +25, the P-score increased for 10 or more trials continuously or in at least 8 out of 10 trials.
Downward type: Games were classified as this type if the P-score kept decreasing and the game ended when the P-score reached −25, the P-score decreased for 10 or more trials continuously or in at least 8 out of 10 trials.
Repetition type: Games were classified as this type if repetitive behaviors were observed in 10 or more consecutive trails.
Unspecified type: Games were classified as this type if the shape of the graph did not fit any of the above defined types.

In the ASP group, 10 out of 29 (34.5%) subjects were classified as upward type, only 2 (6.9%) as downward type, and 5 (17.2%) as repetition type. The characteristic result of this group was the immediate increase of the P-score compared to the control group (21.4% as upward type), in which the rising of the P-score was observed after several trials, suggesting that they selected Card 1 more frequently after the establishment of a certain amount of mutual reliance. Only 2 in the ASP group (6.9%) demonstrated downward type, whereas 8 of the control group (28.6%) were grouped in this type. Both downward type ASP cases answered that they just liked “2,” and one said even numbers were better than odd numbers. This reason was different from that in the control group, which seemed to be related to the dilemma in game theory. Five out of 29 in the ASP group demonstrated some repetition in their results. None of the subjects in the control group was classified as repetition type. This result might be related to one of the core symptom domains of ASP, i.e. repetitive behaviors. These results suggest that the subjects with ASP might be free of the dilemma that commonly arises among two persons because of social deficits in ASP subjects.

### 3. Post-PD Game Interview with the Subjects

Soon after the PD game terminated, the investigator asked the subjects what they were thinking during the game. Although they were instructed to make card selections with the aim of increasing their score, 19 out of 28 (67.9%) control subjects answered that they considered this game a competition, were acutely aware of the study investigator, and tended to make selections in order to win the match. On the other hand, 17 out of 29 (58.6%) ASP subjects answered that they paid attention to the hidden conditions that end the game or the latent rules of the selection behavior of the investigator. In this respect, we speculate that the ASP subjects are prone to have interests in the structure of the game itself or the concealed rules of the behavior of the confronting person.

## Discussion

Using a modified PD game with a variable payoff matrix, we found that subjects with ASP had higher P-scores and total scores compared to control subjects. Fluctuation in P-scores revealed that the ASP group had fewer cases of continuous decrease in P-score compared to controls. This was thought to be due to ASP subjects’ preference for the number 2 itself, rather than to enhance their chance of winning the competition.

Dawes defined social dilemma as the condition that consists of the following two properties: (a) the payoff to each individual for defecting behavior is higher than that for cooperative behavior, regardless of how the other members behave, (b) all individuals in society receive a lower payoff if all defect than if all cooperate [37].

When this condition of social dilemma is observed between 2 subjects, as has been observed among prisoners, it is called prisoner’s dilemma (PD). For example, 2 suspects were arrested for case X whose penalty was 7 years of prison. The police do not have enough evidence to also convict the two subjects for another case Y. If the suspects are convicted for both case X and Y, the total penalty would be full 15 years of prison (8 more years for case Y). Each suspect is investigated separately for case Y and offered a similar deal. If both of them remain silent, they will be sentenced 7 years of prison for only case X. However, if suspect A betrays suspect B by testifying of B’s involvement in case Y, and B keeps silent in another room, A (the betrayer) could have his sentence reduced for both case X and Y from 15 years to 10 years as a reward for his confession. Furthermore, if both A and B confess for case Y, they could each receive a five-year prison time reduction. Each prisoner must choose either to betray the other or be cooperative by remaining silent. The PD game simulates this condition [38].

Pruitt et al indicated that the following conditions are necessary for the development of a reliable relationship. Cooperative behavior often requires a long period of thinking to establish and maintain a relationship of mutual trust. Three perceptions are needed to reach to this goal: (a) dependence on the other (i.e., a recognition of the importance of the other’s cooperation); (b) pessimism about the likelihood that the other can be exploited (i.e., doubting that the other will cooperate unilaterally for a period of time); and (c) insight into the necessity of cooperating with the other in order to accomplish his purpose [39].

There are several studies on cooperative behavior based on mutual trust [27]. Downs et al. investigated the characteristics of cooperative behaviors in high-functioning autism by using the PD game developed by Matsumoto et al. [40]. In terms of cooperative behavior, emotional understanding, and aloof behavior, the autism group was superior to the ADHD group. However, no significant differences have been observed between autism and normal controls with typical development [26].

Sally et al. performed three types of strategic games (Prisoner’s Dilemma, Dictator and Ultimatum) and investigated the relationship between mentalizing, the ability to understand the mental state of oneself and others, and these games in 18 subjects with ASP and normal children [27]. The results demonstrated that the difference between ASP subjects and normal controls was less than previously expected, suggesting that in these games mentalizing skills were not always necessary.

The impaired mentalizing system has been reported to be the cause of the poor social and communication skills in subjects with PDD. Hill et al. investigated the relationship between mentalizing and decision-making in adult subjects with PDD by using the PD game [28]. The behavioral choices of both PDD subjects and normal controls on the PD game showed no group differences between choices made to cooperate or compete. They also conducted a semi-structured interview after the completion of the PD game and found that the thought processes that accompanied behavioral choices in the ASP subjects were both quantitatively and qualitatively similar to that of normal controls. These results suggest that mentalizing skills are not involved in developing a strategy in the PD game. It has been reported that this result can be observed even when human and computer opponents play the PD game. These findings demonstrate that mentalizing is not necessary for altruism in the situation of a social dilemma.

In the interview after the PD game in our study, the control group tended to answer that they tried their best to increase the score, whereas the ASP group was apt to be preoccupied with a specific number, certain rules of selection, elucidation of the hidden conditions to terminate the game, or investigation of the rules of the behavioral choices by the opponent.

In the control group, as a result of excessive pursuit of their own interests, the total score and P-score at the end of the game were lower, and the shape of the graph of P-score fluctuation was the downward type, although some degree of compromise was observed. The significantly higher rate of concession in the classification of selection behaviors demonstrated a certain extent of cooperative attitude towards the opponent in the control group. These results suggested that control subjects had some emotional awareness of the other player.

On the other hand, the subjects with ASP appeared to care less about the intentions of their opponents and imitated their behaviors unconsciously. The higher rate of the upward type fluctuation of P-score suggested that ASP subjects could be less affected by the conflicts that commonly arise among players. Although the upward fluctuation of P-score was observed not only in the ASP group but also in the control group, there was a difference in the fluctuation of the P-score. In the control group, the P-score started rising after several trials suggesting that more frequent selection of cooperative behavior contributed to the establishment of a certain degree of mutual trust. In the ASP group, the P-score increased soon after the beginning of the game suggesting that this result was related to the perseverative behavioral characteristics of ASP. Similar results were observed in the downward type of the P-score fluctuation. Two subjects with ASP whose results were classified in this type were preoccupied by their favorite number and which lead to the repetitive selection of card 2 which represented betrayal. These behaviors may be due to the behavioral characteristics of ASP, i.e. restricted, repetitive patterns of behavior, interests, and activities. As a consequence of these behaviors, we hypothesize that the ASP group was not affected by the dilemma and ultimately achieved higher scores compared to the control group.

In the extreme-male-brain theory of autism proposed by Baron-Cohen, in the mind of a person with autism, systemizing is supposed to be predominant over empathizing [41]. The answers of ASP subjects in the post-game interview are consistent with this theory in terms of lower awareness of others and higher interest in the latent rule behind the game. PDD subjects have difficulties speculating intentions of others. The results of this present study demonstrate characteristic features of cognition in PDD that are distinguished as logical thinking based on a prominent systemizing trait.

Regarding the analysis of the graphs of P-score fluctuation, the repetition type was observed exclusively in PDD group. This result could be related to the one of the essential clinical features of PDD, that is, “restricted repetitive and stereotyped patterns of behavior, interests and activities.” In the upward type in the ASP subjects, the P-score abruptly increased and reached +25 which is one of the conditions that terminate the PD game. This result can be explained by the behavioral characteristics of PDD: to preserve a policy once one has decided it and thereby unconsciously repeat stereotyped behavior.

The downward type was observed both in the ASP and control groups at relatively high rates. It has been reported that this type of P-score change can occur when the player pays much attention to the opponent and has a strong competitive spirit to win the game. In such situations, both players select Card 2 to get a higher profit, and as a result, both of them lose their scores.

In the ASP group, only 2 out of 31 subjects fit the downward type graph. However, their strategy reported in the interview after the game was very different from that of the controls. One of them answered that “I like 2” and the other said “even is better than odd.” In other words, their behavior could be explained by preoccupation with a certain number.

In the PD game with a variable factor that we used in the present study, ASP subjects were less likely to be affected by the dilemma compared to the control subjects. As a consequence, the total score and the P-score at the end of the game were higher in ASP subjects than in controls. The repetition of selecting the same number and a certain sort of rule to their behavior is closely related to one of the 3 core clinical characteristics of ASP.

### Conclusions

It is often difficult to diagnose PDD in the adolescent, especially PDD-NOS which has subthreshold clinical symptoms. The results of the PD game revealed behavioral characteristics of ASP subjects such as little awareness toward others, preoccupation with their interests, and restricted and repetitive patterns of behavior. ASP subjects are apt to pursue their interests without paying attention to those around them. Our results suggest the possibility of the clinical application of the PD game as a diagnostic support tool for PDD. We await further studies with interest.
